# Acoustic pattern evaluation during cementless hip arthroplasty surgery may be a new method for predicting complications

**DOI:** 10.1051/sicotj/2016049

**Published:** 2017-02-13

**Authors:** Itaru Morohashi, Hideaki Iwase, Akio Kanda, Taichi Sato, Yasuhiro Homma, Atsuhiko Mogami, Osamu Obayashi, Kazuo Kaneko

**Affiliations:** 1 Department of Orthopaedic Surgery, Juntendo University Shizuoka Hospital 1129 Nagaoka Izunokuni Shizuoka 410-2295 Japan; 2 Department of Bio-Engineering, Juntendo University Institute of Casualty Center 1129 Nagaoka Izunokuni Shizuoka 410-2295 Japan; 3 Department of Host Defense and Biochemical Research, Tokyo Denki University 5 Senju Asahi-cho Adachi-ku Tokyo 120-8551 Japan; 4 Department of Orthopaedics, Juntendo University 2-1-1 Hongo Bunkyo-ku Tokyo 113-8421 Japan

**Keywords:** Total hip arthroplasty, Cementless stem, Intraoperative fracture, Subsidence, Sound analysis

## Abstract

*Background*: Although surgeons must perform implantation of the cementless stem during total hip arthroplasty (THA) without complications, assessment is left to the surgeon’s intuitive judgement, which could contain inter/intra-observer bias variety. We therefore asked (1) whether the sound created during the stem implantation could be evaluated objectively and (2) whether those sounds are correlate to the complication specific to the cementless stems. Our hypothesis is that the sounds produced during stem insertion could be quantified and related to the complications.

*Patients and method*: In 71 THAs, we quantified the sound produced during stem insertion and investigated the relationship between these sounds and the occurrence of intraoperative fracture and subsidence.

*Results*: The sound data were divided into two patterns: Patterns A and B. The difference between the peak value (dB) at the most common frequency (near 7 kHz) and the second most common frequency (near 4 kHz) of strikes during the final phase of implantation in Patterns A and B showed a significant difference. Adverse events on intraoperative fracture and subsidence were significantly less common in patients with Pattern A than in those with Pattern B (six of 42 hips with Pattern A and 13 of 29 hips with Pattern B, *p* = 0.004). Pattern A in predicting a clinical course without those adverse events was 69.2% and the specificity was 68.4%. Positive and negative predictive values were 85.7% and 44.8%, respectively.

*Conclusion*: The sound generated during stem insertion was quantified. Those sound patterns were associated with complications.

## Introduction

Total hip arthroplasty (THA) has become an increasingly common orthopaedic procedure throughout the world. Cementless THA surgeries have increased in popularity in recent years [1, 2]. However, compared with cemented THA, cementless THA has specific complications, including thigh pain [3], subsidence [4, 5] and intraoperative fracture [6, 7]. Subsidence can cause leg length discrepancy and increased risk of dislocation owing to impingement and loosened soft tissue tension. Early postoperative implant instability and micromotion are also associated with aseptic implant loosening [4, 5, 8]. Intraoperative femoral fractures occur in 3.0%–5.4% of primary THA and in 19.0%–20.9% of revision THA procedures [6, 7, 9, 10]. Intraoperative fractures often require additional osteosynthesis procedures. Keys to successful stem implantation include avoiding malalignment and selecting the proper stem size. Adequate force is needed to achieve firm implantation; however, overzealous implantation can cause femur fractures [7, 11], whereas inadequate force can result in postoperative subsidence. This fine line between excessive and insufficient force is a surgical challenge for surgeons. Although surgeons must perform sufficiently firm implantation without fracturing the femur, assessment of the appropriate force and stem stability is usually left to the surgeon’s intuitive judgement. Based on accumulated experience, surgeons use changes in sound as the stem becomes more stable to empirically aid assessment of fixation; however, a big problem is that this judgement is subjective, which could potentially contain inter/intra-observer bias variety. We therefore asked (1) whether the sound created during the stem implantation could be evaluated objectively and (2) whether those sounds are correlate to the complication specific to the cementless stems. Our hypothesis is that the sounds produced during stem insertion could be quantified and related to the complications specific to cementless stem THA. This prospective study was designed to objectively quantify the sound produced during stem insertion, and to investigate the relationship between these sounds and the occurrence of intraoperative fracture and postoperative subsidence.

## Materials and methods

### Experimental setup and identification of natural oscillation frequency of materials

**Figure 1.**
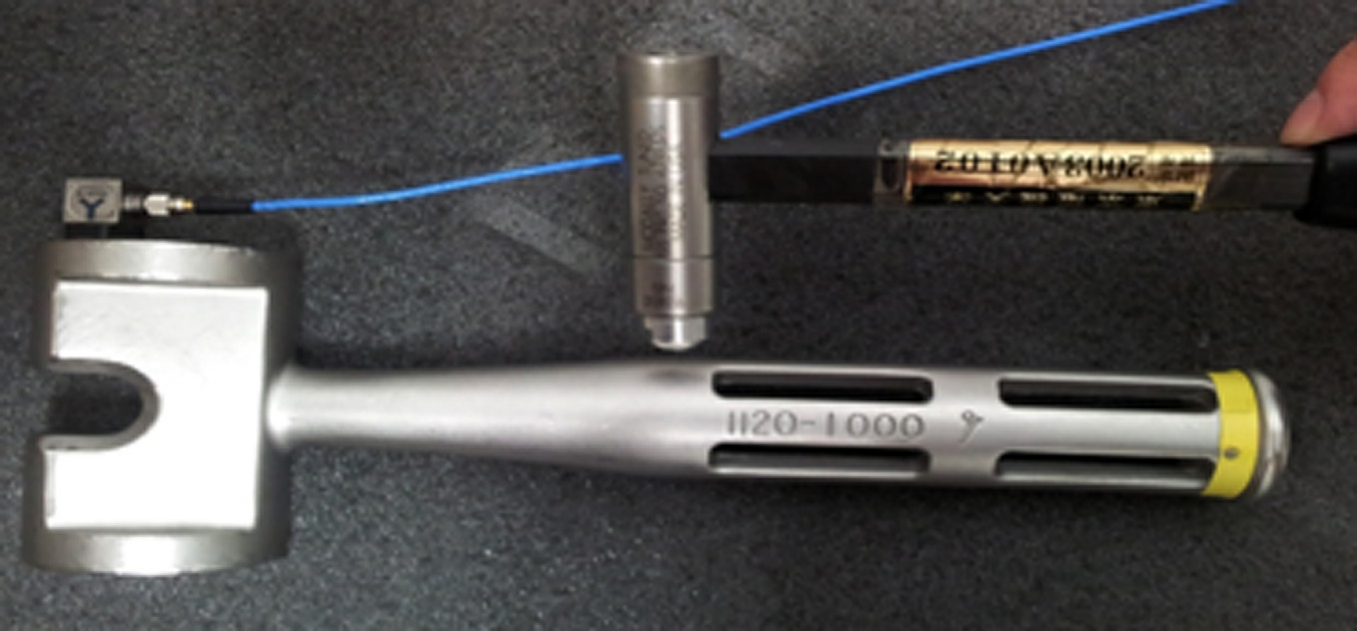
The analysis of the natural oscillation frequency of the materials.

We analysed the natural oscillation frequency of each instrument to determine which instrument created individual sounds during implantation. All objects have a natural oscillation frequency, which can be determined by striking an object with an impact hammer to induce oscillation. We used this method to measure the oscillation frequency of the hammer, stem and impactor (Figure 1).

### Patients

Institutional review board’s approval was obtained before this prospective study. A total of 109 THAs were performed at our hospital between January 2012 and July 2013; all of these were initially included in this study. Exclusion criteria were: (1) refusal to participate in the study; (2) previous osteotomy of the femur; and (3) failed osteosynthesis of the proximal femur. The remaining patients were included in the study. Age, body mass index (BMI), etiology of hip disease affected side and canal-flare index were investigated. A total of 71 hips were included in the analysis. There were 12 male and 59 female patients, with a mean age of 65.8 years (range, 41–86 years) at surgery. Osteoarthritis was diagnosed in 63 hips (88.7%), osteonecrosis in five (7.0%), rheumatoid arthritis in one (1.4%) and femoral neck fracture in two (2.8%). Forty procedures involved the right hip (56.3%); 15 patients had bilateral procedures. The mean body mass index was 24.3 kg/m^2^ (range, 17.7–38.2 kg/m^2^).

### Surgical procedure

Surgeries were performed by two orthopaedic surgeons with more than 10 years of experience. The stem used in all cases was a cementless proximally hydroxyapatite-coated component (Accolade TMZF; Stryker Corporation, Tokyo, Japan). The surgeons used a direct lateral approach in 59 patients, a minimally invasive anterolateral approach in seven patients and a direct anterior approach in nine patients. The stem size was determined by performing a trial insertion of the stem and taking intraoperative radiographs. It was confirmed that the stem was not in a varus position, and that the porous sites on the medial and lateral sides of the implant were in contact with cortical bone. Postoperative rehabilitation involved full weight bearing beginning on the first day after surgery in all cases, when possible.

### Intraoperative sound data collection

**Figure 2.**
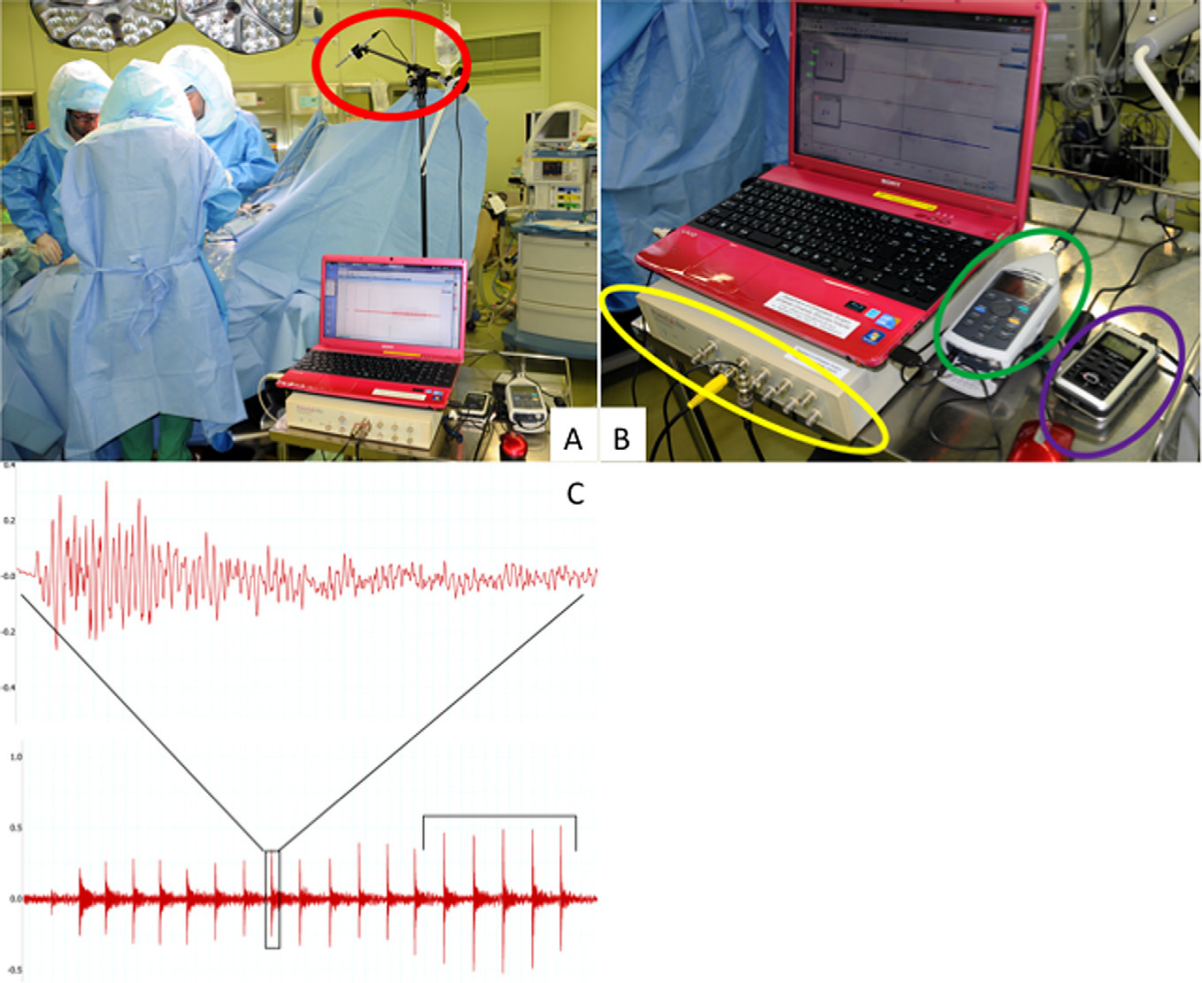
Intraoperative sound data collection. The sound made with the impactor during stem implantation was recorded (A). A PowerLab data logger and performed sampling at 40 k/s was used (B). A sound analysis specialist analysed the data in a blind fashion (C).

We recorded the sounds made with the impactor during stem implantation (implantation sounds) on a data logger (AD Instruments, Tokyo, Japan) with LabChart software (AD Instruments, Tokyo, Japan) (Figure 2A). Fast Fourier transform frequency analysis was performed on the implantation sounds (Figure 2A). A sound level meter (LA-4440; Ono Sokki Technology, Tokyo, Japan) was used during implantation sound recording. We used a PowerLab data logger and performed sampling at 40 k/s (Figure 2B). Analysis involved recording the signal emitted during implantation, adding five of the resulting waves when the stem was subjectively fixed and performing fast Fourier transform analysis of the waveform. A sound analysis specialist analysed the data in a blind fashion (Figure 2C).

### Assessment of postoperative subsidence

Radiographic measurement of subsidence was made on anteroposterior radiographs of the hips in the supine position. A single observer (YH), who was not involved in treatment, manually measured and analysed the measurements with the ruler function on the picture archiving and communication system at our institution (Fujifilm Synapse 3.2.1 SR-356; Fujifilm Corporation, Tokyo, Japan). A previously reported measurement method was used [12]. Radiographs were evaluated immediately after surgery and two weeks later. A difference ≥1 mm was defined as subsidence in this study.

### Assessment of intraoperative femoral fracture and use of the bone model

Intraoperative femoral fracture was assessed during surgery with careful attention and intraoperative X-rays. However, because intraoperative fracture was rare, we also used a bone model for analysis. Polyurethane femur models (1103; Sawbones Company) embedded in silicone resin were used. The stem was implanted to simulate actual surgery until visible bone fracture. Sound data were collected as described above.

### Statistical analysis

Baseline characteristics are expressed as means (standard deviation). Student’s *t*-test or the Welch test was used for continuous variables. Pearson’s chi-squared test and Fisher’s exact test were used for dichotomous variables. Values of *p* < 0.05 were considered statistically significant, and all tests were two-sided. Data were statistically analysed with IBM SPSS Statistics for Macintosh (Version 22.0; IBM, Armonk, NY, USA).

## Results

### Identification of the natural oscillation frequency of each material

**Figure 3.**
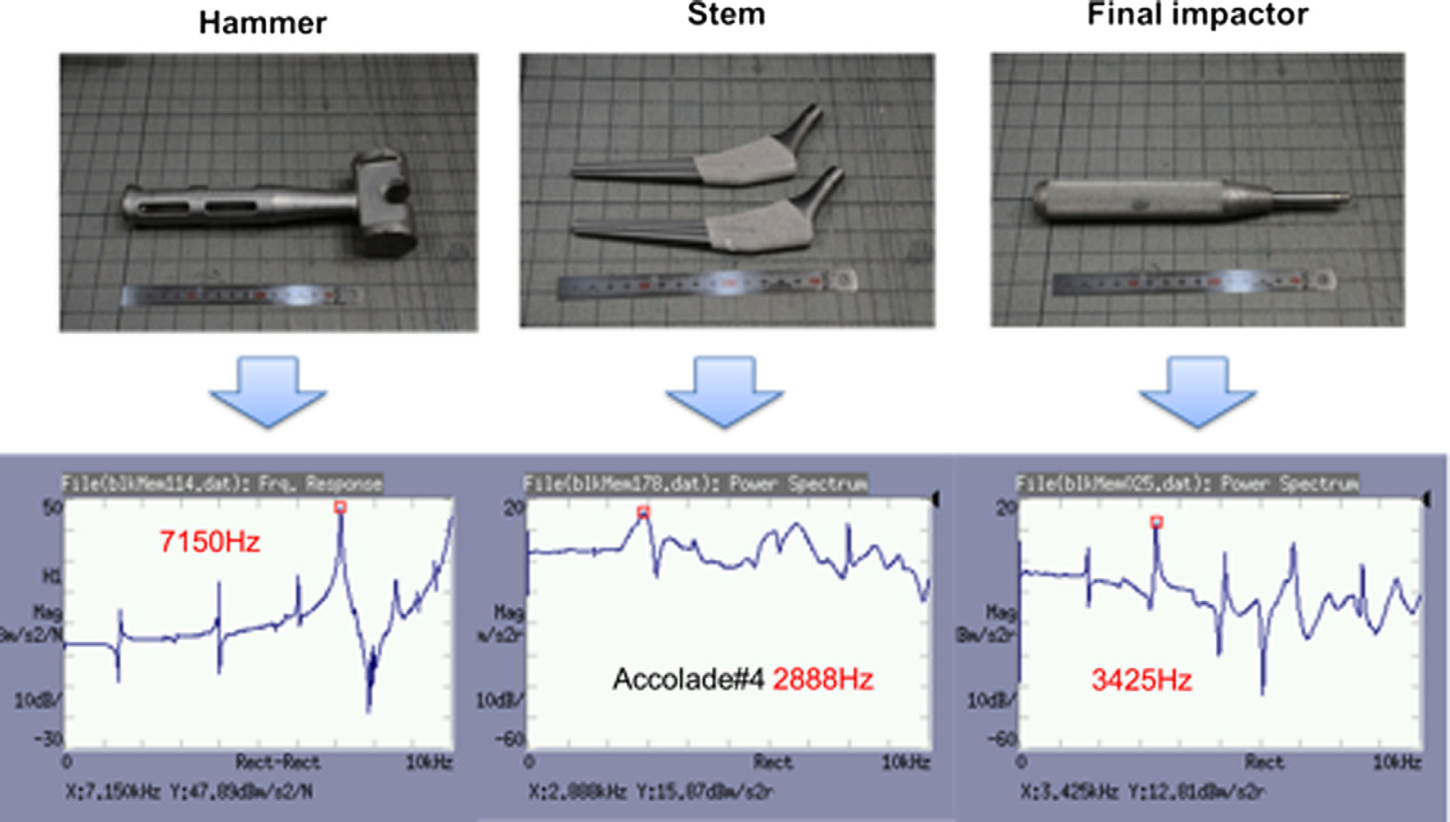
Identification of the natural oscillation frequency of each material.

The natural oscillation frequency of the hammer was approximately 7 kHz, that of the stem (Accolade TMZF #4; Stryker Corporation, Tokyo, Japan) was 3 kHz and that of the final impactor was 3.1 kHz (Figure 3).

### Sound data analysis

**Figure 4.**
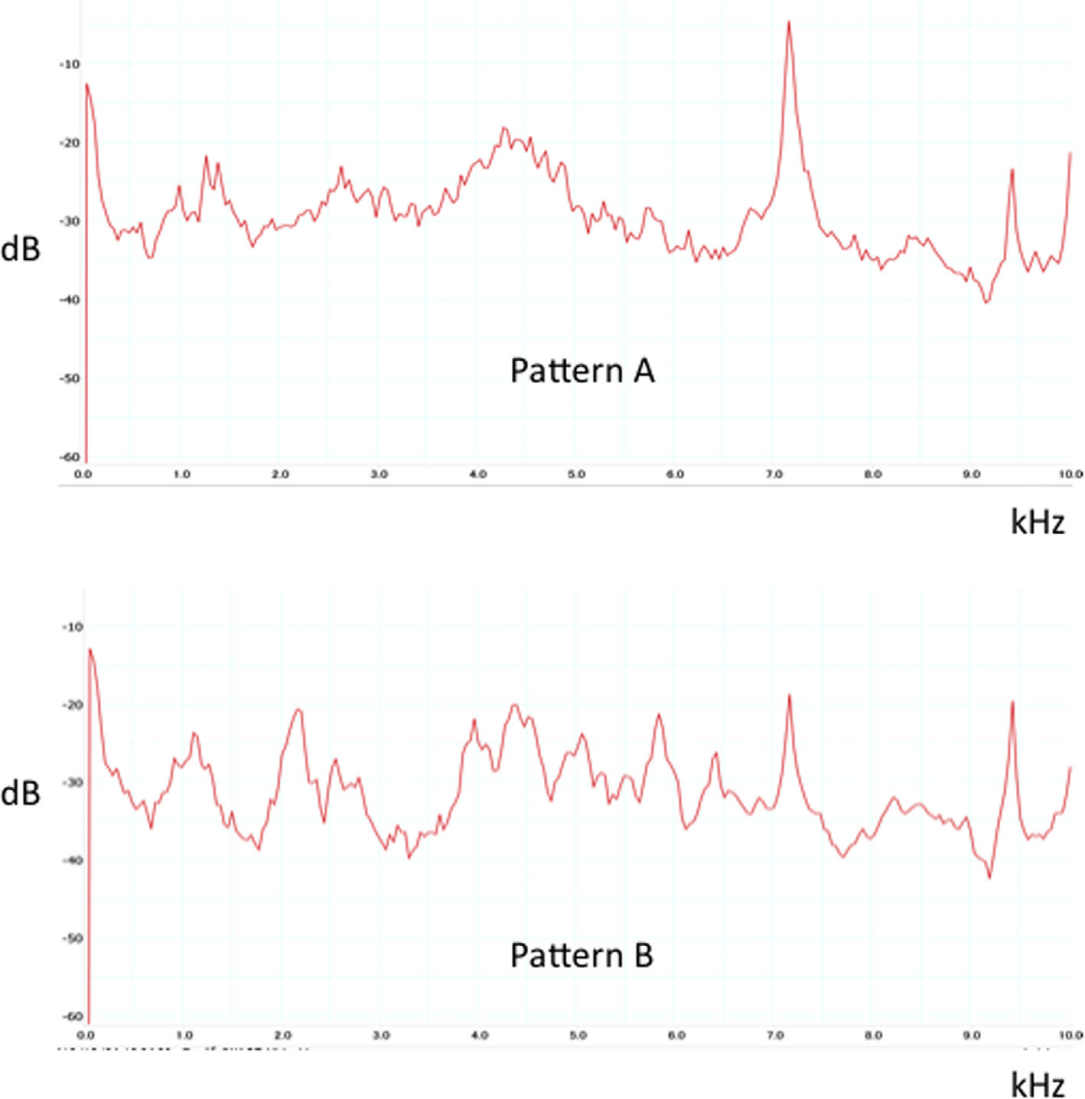
Typical sound data of Patterns A and B. Pattern A, in which frequencies near 7 kHz were more accentuated than other frequencies as the implantation progressed, and Pattern B, in which there was no accentuation of frequencies near 7 kHz through completion of implantation.

Based on the evaluation of the sound specialist in a blind fashion, the data were divided into two patterns: Pattern A, in which frequencies near 7 kHz were more accentuated than other frequencies as the implantation progressed, and Pattern B, in which there was no accentuation of frequencies near 7 kHz through completion of implantation (Figure 4). The difference between the peak value (dB) at the most common frequency (near 7 kHz) and the second most common frequency (near 4 kHz) of the last five strikes during the final phase of implantation was assessed in both Pattern A and Pattern B. Pattern A had a significantly greater difference in peak value between the two major frequencies than Pattern B (Table 1).

**Table 1.** Sound data analysis showed that pattern A had a significantly greater difference in peak value between the two major frequencies than Pattern B. Pattern A could predict good clinical course.

Parameters	Pattern A	Pattern B	*p* value
Age (*SD*)	58.4 (12.7)	54.2 (9.4)	0.123
BMI (*SD*)	24.5 (4.3)	23.3 (3.6)	0.128
Canal-flare index (*SD*)	3.6 ± (0.7)	3.9 (0.8)	0.16
Δ of the dB between two major frequencies	10.2 ± 5.3	5.1 ± 6.8	0.001**
Postoperative subsidence	6/48	11/27	0.013**
Clinical course without adverse events*	36/42	16/29	0.004**

* Adverse events; intraoperative fracture, subsidence.

** Significant difference.

*SD*: Standard deviation.

### Assessment of the relationship between the sound pattern and clinical outcomes

Pattern A was found in 42 of 71 patients and Pattern B in 29 of 71 patients. Intraoperative fracture occurred in two hips at the time of stem implantation. Until the crack appeared, these patients demonstrated Pattern A; after fracture, they demonstrated Pattern B. During the implantation experiments with the bone model, the model demonstrated a waveform similar to Pattern A until immediately before a crack appeared, then changed to Pattern B immediately after fracture.

Adverse events on intraoperative fracture and postoperative subsidence were significantly less common in patients with Pattern A than in those with Pattern B (six of 42 hips with Pattern A and 13 of 29 hips with Pattern B, *p* = 0.004) (Table 1). The sensitivity of Pattern A in predicting a clinical course without those adverse events was 69.2% and the specificity was 68.4%. Positive and negative predictive values were 85.7% and 44.8%, respectively (Table 2). Excluding the two patients who experienced intraoperative fracture, there was a significant difference in the incidence of subsidence between Patterns A and B (6 of 42 hips in patients with Pattern A and 11 of 27 hips in those with Pattern B, *p* = 0.013) (Table 1). The sensitivity of Pattern B in predicting subsidence was 64.7% and the sensitivity was 69.2%. Positive and negative predictive values were 40.7% and 85.7%, respectively (Table 2).

**Table 2.** Diagnostic accuracy of the acoustic analysis using Patterns A and B.

	Value	95% confidence interval
Pattern B for the subsidence		
Sensitivity	0.65	0.443–0.812
Specificity	0.69	0.628–0.746
PPV	0.41	0.279–0.511
NPV	0.86	0.775–0.924
Pattern A for the clinical course without adverse events*		
Sensitivity	0.69	0.623–0.747
Specificity	0.68	0.493–0.833
PPV	0.86	0.771–0.924
NPV	0.45	0.323–0.546

PPV: positive predictive value, NPV: negative predictive value.

* Adverse events; intraoperative fracture, subsidence.

## Discussion

Prevention for the complication specific to cementless stem in THA such as intraoperative fracture and postoperative subsidence is very important. So far, surgeon’s subjective judgement is largely contributed in it, and no objective evaluation exists. In the present study, the sound generated during stem insertion was quantified, and two sound patterns were identified. Those distinct patterns were associated with intraoperative fracture and postoperative subsidence, complications specific to cementless stems. Our results highlight the possibility of using sound analysis to assess the risk of complications intraoperatively.

Our method of analysing differences in the peak sound value of each material is easily applied for assessment during surgery. The sounds in a natural environment consist of various natural oscillation frequencies. When an object is struck by another material or moves itself, vibrations result, which transmits through space as a frequency, resulting in an audible sound. Sounds that arise when one object strikes another are composed of the frequencies of the striking and the struck object. In this study, we assumed that the natural oscillation frequency of the hammer becomes the prominent frequency, compared with those of the stem and the final impactor, as stem movement decreases with adequate fixation in the femur. When the frequencies of each material are mixed, so that no prominent hammer frequency is present, the stem remains movable in the femur, increasing the risk of postoperative subsidence as a result of inadequate fixation. Our results also suggest that when intraoperative fracture occurs, the sound pattern changes from A to B, possibly indicating that excessive force was applied after adequate fixation.

We believe that this method of acoustic evaluation is an easy, reliable way to ensure the safe execution of THA with cementless stems. In engineering, there are two methods for assessing the interior of materials: acoustic analysis similar to our method, which involves analysing the frequency of a sound produced by striking an object [13, 14], and vibration analysis, which involves initiating vibrations in an object and analysing the frequency of those vibrations [15–18]. Acoustic analysis involves recording a sound produced by the target object. The advantage of this method is that no equipment needs to be attached to the target object; the disadvantage is that ambient sounds may be inadvertently included in the recording. Oyama et al. applied the method of acoustic assessment in orthopaedic surgery, emphasizing that this method of intraoperative assessment of the amount of stem implantation was easier than other methods and could be performed in real time [19]. They recognized it as a more indirect assessment than the vibration method and considered it supplementary information to assess stem fixation [19]. In their study, subjects were divided into two groups: a convergence group, in which the frequency distribution of three impact sounds during stem implantation was uniform, and a non-convergence group, in which the distribution was not uniform. They reported that there was a relationship between the sounds and the percentage of medullary cavity occupied by the stem. Those data are compatible with our results.

In contrast, vibration analysis involves installing an accelerometer on the target object and measuring and analysing the vibration acceleration. Although this method has the advantage of yielding highly precise analytical results, it has the disadvantage of requiring that an accelerometer be attached to the target object. This issue is a serious disadvantage in joint replacement surgery, because sterility is essential to avoid infection. Many studies have reported the advantages of vibration methods [15–18]. Rosenstein et al. performed postoperative vibration analysis and reported that they were able to assess the stability of the inserted femur stem [15]. In addition, Pastrav et al. performed excitation of the femur stems with an excitation device (random excitation using 0–12.5 kHz white noise). That group determined the frequency-response function (acceleration) and used the impedance head during each stage of placement to exclude the input (excitation force). When they investigated the relationship between changes in the peak frequency expressed during this process and stem implantation, they found almost complete correlation between the frequency-response function and the two final stages of implantation [18]. These types of vibration experiments are commonly used to gain an understanding of the innate oscillation frequency of a structure or the dynamic characteristics of the nature of attenuation. The vibration system made up of the femur and stem is based on the attenuation characteristics and combined rigidity of both objects, which change is based on changes in the status of contact between the femur and the stem.

There are several limitations to this study. First, the impact technique was not standardized. Thus, it is possible that the quality and quantity of sound (dB) created by the hammer depended on the surgeon’s technique. However, this was a clinically based study; thus, the analysis should include ambiguous human performance. In addition, there were no outlier values of the sounds (dB) in this study. Second, the diagnosis of intraoperative fracture was not made with an accurate test such as computed tomography. It is possible that occult fracture of the femur occurred. However, we believe that this limitation had minimal effect, because every patient immediately started full weight bearing without clinical adverse events such as the need for revision surgery. Third, sound analysis in this study was performed after surgery. Future studies are needed to develop the technique further, including studying the possibility of real-time assessment. As a conclusion, we demonstrated that the sound generated during stem insertion was quantified, and two sound patterns were identified. Those distinct patterns were associated with intraoperative fracture and postoperative subsidence, complications specific to cementless stems. Our results highlight the possibility of using sound analysis to assess the risk of complications intraoperatively.

## Compliance with ethical standards

### Conflict of interest

The authors declare that they have no conflict of interest.

### Ethical approval

All procedures performed in studies involving human participants were in accordance with the ethical standards of the Institutional and/or National Research Committee and with the 1964 Helsinki Declaration and its later amendments or comparable ethical standards. The study was approved by the Medical Research Ethics Committee at our university.

### Informed consent

Informed consent was obtained from all individual patients in this study.
